# Poststroke psychosis: a systematic review

**DOI:** 10.1136/jnnp-2017-317327

**Published:** 2018-01-13

**Authors:** Helle Stangeland, Vasiliki Orgeta, Vaughan Bell

**Affiliations:** 1 Division of Clinical Neuroscience, Oslo University Hospital, Oslo, Norway; 2 Division of Psychiatry, University College London, London, UK

**Keywords:** stroke, neuropsychiatry, hallucinations, schizophrenia

## Abstract

A preregistered systematic review of poststroke psychosis examining clinical characteristics, prevalence, diagnostic procedures, lesion location, treatments, risk factors and outcome. Neuropsychiatric outcomes following stroke are common and severely impact quality of life. No previous reviews have focused on poststroke psychosis despite clear clinical need. CINAHL, MEDLINE and PsychINFO were searched for studies on poststroke psychosis published between 1975 and 2016. Reviewers independently selected studies for inclusion, extracted data and rated study quality. Out of 2442 references, 76 met inclusion criteria. Average age for poststroke psychosis was 66.6 years with slightly more males than females affected. Delayed onset was common. Neurological presentation was typical for stroke, but a significant minority had otherwise ‘silent strokes’. The most common psychosis was delusional disorder, followed by schizophrenia-like psychosis and mood disorder with psychotic features. Estimated delusion prevalence was 4.67% (95% CI 2.30% to 7.79%) and hallucinations 5.05% (95% CI 1.84% to 9.65%). Twelve-year incidence was 6.7%. No systematic treatment studies were found. Case studies frequently report symptom remission after antipsychotics, but serious concerns about under-representation of poor outcome remain. Lesions were typically right hemisphere, particularly frontal, temporal and parietal regions, and the right caudate nucleus. In general, poststroke psychosis was associated with poor functional outcomes and high mortality. Poor methodological quality of studies was a significant limitation. Psychosis considerably adds to illness burden of stroke. Delayed onset suggests a window for early intervention. Studies on the safety and efficacy of antipsychotics in this population are urgently needed.

## Introduction

When considered individually, stroke and psychosis are known to be among the most disabling of all health conditions.^1^ Globally, approximately 16 million people have their first stroke each year of which approximately 5.7 million people die and 5 million are left with long-term disabilities.^2^ Neuropsychiatric symptoms following stroke occur in at least 30% of stroke survivors and are a major predictor of poor outcome,^2 3^ and the particular combination of stroke and psychosis is considered to be among the most serious of the poststroke syndromes.^3–5^ Neuropsychiatric symptoms are often underdiagnosed and undertreated after stroke,^2 3^ and poststroke psychosis seems to have attracted remarkably little research attention to inform evidence-based practice despite its serious nature and treatment implications. Perhaps as a result, psychosis is often excluded from treatment guidelines for poststroke psychiatric management.^6^

Currently, there is no systematic review on poststroke psychosis, despite a clear need for a summary of existing evidence. Consequently, we completed a preregistered systematic review of poststroke psychosis to synthesise evidence on prevalence, diagnostic procedures, aetiology, clinical characteristics, treatment and outcome.

## Methods

A review protocol specifying the rationale and planned methods for the review was registered at PROSPERO. Registration number CRD42015021987; http://www.crd.york.ac.uk/PROSPERO/display_record.asp?ID=CRD42015021987.

The systematic review was conducted in accordance with the Preferred Reporting Items for Systematic Reviews and Meta-Analyses (PRISMA) guidelines.

### Eligibility criteria

Stroke was defined as any form of stroke of either ischaemic or haemorrhagic aetiology, including transient ischaemic attack. Because of inconsistency in the use of diagnosis for poststroke psychosis, studies were selected on the basis of specific criteria independently assessed by the study authors: psychosis was defined as the presence of hallucinations or delusions. Hallucinations were included if they were experienced in full consciousness and were considered to be complex and accompanied by reduced or absent insight. Where the hallucinations were only present at night (eg, in peduncular hallucinosis) or could be explained by other non-specific causes, such as delirium, the studies were excluded. Delusions were included if they were experienced in full consciousness and could not be better explained by somatoparaphrenia and anosognosia for neurological impairment. Although somatoparaphrenia and anosognosia are sometimes classified as delusions, they are typically not included under the definition of poststroke psychosis^7^ and were therefore excluded.

Studies including participants with pre-existing neurodegenerative disorders (eg, dementia) that would make differential diagnoses especially challenging were excluded from the review. All study types from single-case studies to randomised controlled trials were considered eligible for inclusion. Case reports or case series were only included if they reported a clinical assessment of stroke pathology alongside MRI or CT scan results to assess lesion location.

### Information sources, search and synthesis

Cumulative Index to Nursing and Allied Health Literature (CINAHL), MEDLINE and PsychINFO were electronically searched from January 1975 to December 2016. Two reviewers independently screened studies for inclusion and read the full text of selected articles to make the final inclusion decision, extracted data and rated the quality of all included studies. Further details on the search strategy, study criteria, definitions used, data extraction and quality assessment are presented in the online supplementary information.

10.1136/jnnp-2017-317327.supp1 Supplementary file 1

## Results

**Figure 1:**
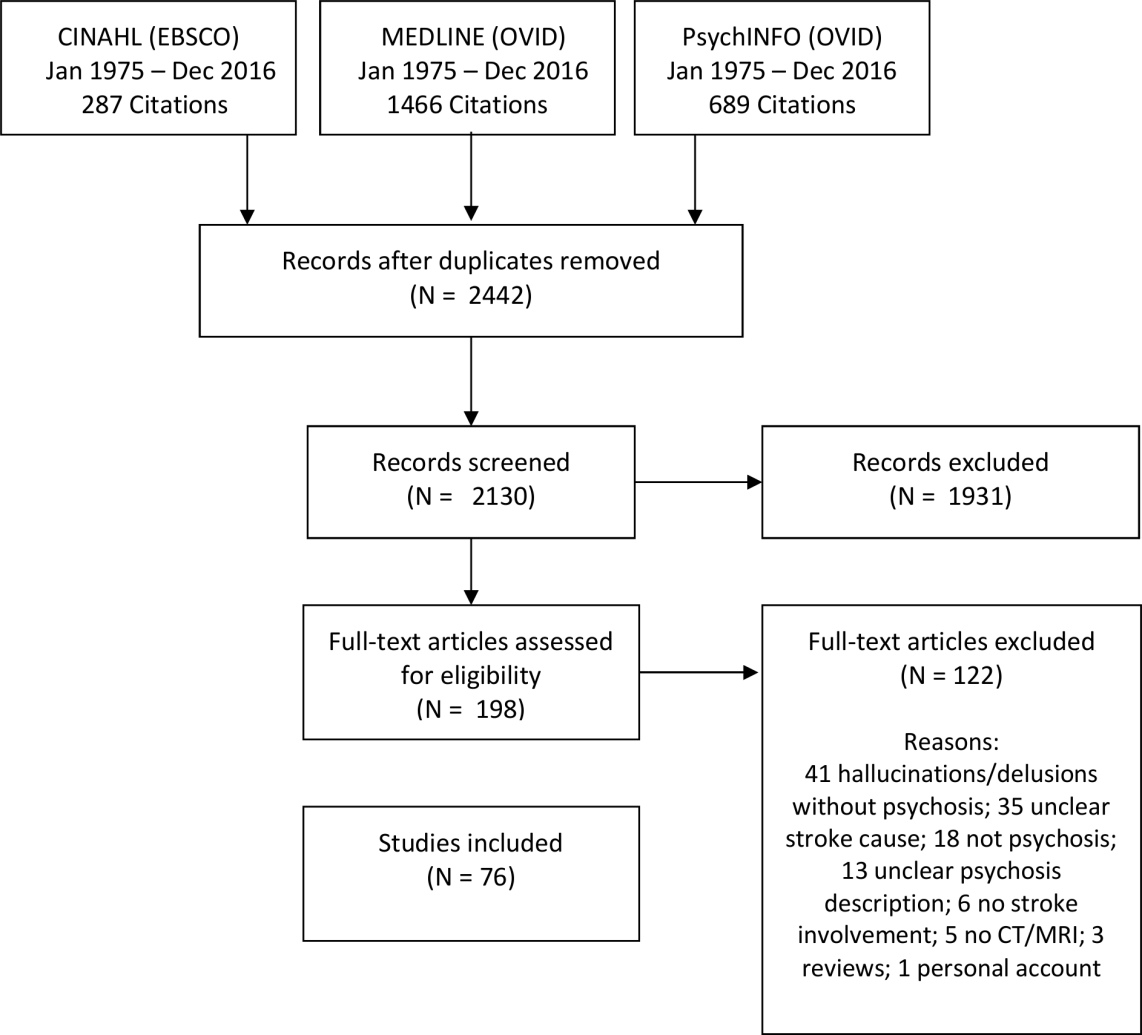
Flow diagram for literature search and selection.

A total of 2442 articles were identified through database searching. After removal of duplicates and clearly irrelevant articles, 198 were identified as potentially eligible. Out of these, 122 studies were excluded as not meeting inclusion criteria. A total of 76 studies, published between 1984 and 2016, were included in the final review. Of the included studies, 1 was a prospective cohort study, 3 were cross-sectional studies, 3 were retrospective cohort studies, 11 were case series and 58 were case reports. Due to the low number of controlled studies, meta-analysis was only possible for prevalence data. Reasons for exclusion and the full references of included studies can be found in the online supplementary information. A flow diagram of the results of the search is presented in figure 1.

### Presenting characteristics

In total, individual details for 264 patients with poststroke psychosis were identified across relevant studies included in the review. Among the 68 studies that reported relevant demographic data (n=170), mean age was 66.6 years (SD=16.59; range 8–96). In terms of gender, 79 (46.5%) of the patients were female (mean age*=*65.9 years; SD=17.13) and 91 (53.5%) of the patients were male (mean age=67.1 years; SD=16.01).

Type of stroke was reported in 67 studies: 150 patients (79.8%) were reported with ischaemic strokes and 34 (18.1%) with haemorrhagic strokes. Four patients were reported with transient ischaemic attack (2.1%). Presenting neurological symptoms were reported in 60 out of the 76 studies, reporting on 80 patients in total (30.3%), with the most common being left-sided weakness (10.6% of patients), headache (4.2%) and slurred speech (3.8%). Other common presenting symptoms included left-sided neglect, left-sided decreased vision or blindness, left facial weakness and confusion. A total of 8% of patients were reported with otherwise ‘silent strokes’ with psychosis being the only prominent presenting symptom and stroke only confirmed on subsequent investigation.

In a retrospective cohort study on all stroke patients in Western Australia, Almeida and Xiao^8^ reported a mean time to onset for psychosis of 6.11 months poststroke. Specifically focusing on delusions, Kumral and Oztürk^9^ reported time to onset from point of acute stroke admission and reported an average onset of 2 days (range 1–3 days) with brief duration (mean 13.1 days, range 5 days–1 month), although the inclusion of two patients with disorientation and several with agitation raise the possibility of inadequate screening for delirium.

Delusional disorder (delusion without the presence of hallucinations) was the most common diagnosis, which was reported in 42 studies and included 82 patients (31.1%) of which 15 studies (7.8% of patients) only reported a delusional misidentification syndrome. In a total of 16 studies (8.3% of patients) schizophrenia-like psychosis (presence of hallucinations and delusions) was reported, and in 5 studies (1.9% patients) mood disorders with psychotic features were reported. Unspecified psychosis or psychotic features were reported in 11 studies and involved 153 patients (58%). One case study reported delusional confabulations and a further case study reported distorteidolias (complex perceptual distortions).

For those with delusions, themes included persecutory delusions, present in 40 patients (15.2%), Othello syndrome/delusional jealousy present in 15 patients (5.7%), reduplicative paramnesia present in 15 patients (5.7%) and somatic delusions present in 14 patients (5.3%). In 13 patients (4.9%), monothematic delusions were described. The most common types of hallucinations were auditory, reported in 14 patients (5.3%), and visual, present in 7 patients (2.7%). A small number of the case reports described less common delusions, including Fregoli, Cotard and Capgras syndromes. Full details of all individual case reports are presented in online table 1 of the supplementay information.

### Lesion location

Information concerning lesion location was available in 67 studies reporting a case study or case series and included 134 patients (50.8%). A total of 106 (40.2%) patients were reported to have lesions in the right hemisphere, whereas only 19 (7.2%) patients were reported to have lesions in the left hemisphere, and 9 (3.4%) patients were reported to have bilateral lesions. The regions that were most often affected included the right frontal, temporal and parietal regions, as well as the right caudate nucleus. The right frontal region was affected in 30 (11.4%) patients, the right temporal region was affected in 26 (9.8%) patients and the right parietal region was affected in 40 (15.2%) patients. The right caudate nucleus was affected in 14 (5.3%) patients. The most common affected artery was the right middle cerebral artery, which was reported to be affected in 22 (8.3%) patients.

Of the case series included in this review, six specifically set out to explore lesion location and its potential role in psychosis. Of these, two studies included patients with schizophrenia-like psychosis,^10 11^ three included patients with delusions only^9 12 13^ and one included patients with delusional misidentification syndromes only.^14^ Of the two studies that looked at schizophrenia-like psychosis after stroke, one found that the right hemisphere cortical lesions were the most common site of lesion, which predicted delusions when present with pre-existing subcortical atrophy,^10^ while the other found that the right inferior frontal gyrus and underlying white matter was the most common lesion location.^11^ Among the three studies that included patients with delusions only, one found that right posterior temporoparietal lesions were most common,^9^ one found lesions to the right central gyri and frontotemporal regions to be the most common^12^ and one reported delusions associated with the right caudate nucleus.^13^ Finally, the study that included patients with delusional misidentification syndromes found an association between the right hemisphere and reduplicative paramnesia.^14^

### Prevalence and incidence rates

Four systematic studies reported prevalence rates of delusions,^9 15–17^ and three studies reported prevalence of hallucinations.^15–17^ Of these three studies, Kumral and Oztürk^9^ reported clinically diagnosed delusions in a sample of acute stroke patients. Buijck *et al*^15^ reported prevalence rates for delusions and hallucination on admission and discharge in a sample of geriatric patients admitted for stroke rehabilitation, van Almenkerk *et al*^16^ reported prevalence rates of delusions and hallucinations in a sample of institutionalised stroke patients and Wong *et al*^17^ reported on delusion and hallucination prevalence in hospitalised stroke patients 3–6 months after stroke. Buijck *et al*,^15^ Almenkerk *et al*^16^ and Wong *et al*^17^ used the Neuropsychiatric Inventory Questionnaire to measure delusions and hallucinations. To estimate cross-sectional prevalence, only admission prevalence was included from Buijck *et al*.^15^

**Figure 2:**
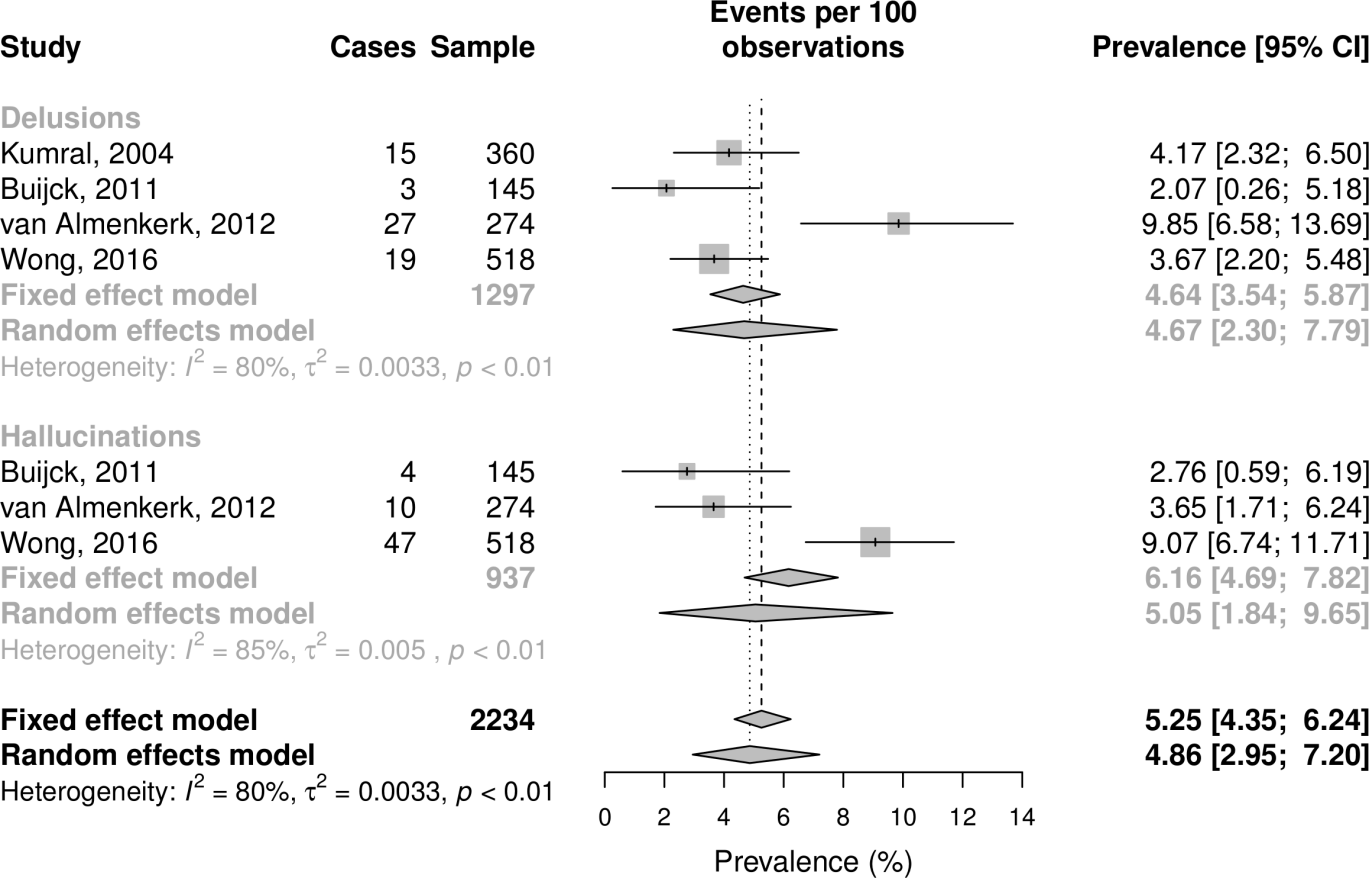
Forest plot of poststroke delusion and hallucination prevalence.

A meta-analytic summary of these studies are shown in figure 2, and full details of the procedure are reported in the online supplementary material. Using the more conservative random effects model that better accounts for study heterogeneity, estimated prevalence rate for delusions was 4.67% (95% CI 2.30% to 7.79%), and the estimated prevalence rate for hallucinations was 5.05% (95% CI 1.84% to 9.65%) with a combined prevalence of 4.86% (95% CI 2.95% to 7.20%). Due to differences in assessment, study design and stroke population in Kumral and Oztürk,^9^ we also conducted the analysis with this study removed (full results reported in online supplementary material figure S1), which led to an almost identical prevalence estimate for delusions but with widened CIs (4.83%, 95% CI 1.53 to 9.72).

Only one study reported a long-term incidence rate of poststroke psychosis. Almeida and Xiao^8^ looked at all stroke patients from Western Australia finding a cumulative incidence rate of 6.7% for psychosis in people without prior psychiatric disorder during the 12 years after first stroke (1.11 per 1000 person-years). The same study also reported that among all stroke survivors who subsequently received a psychiatric diagnosis after 24 months, psychotic disorders were present in 12.2%.

Additionally, one study that compared measures of psychological distress between patients 1 year after stroke and a group of matched controls found that mean scores of paranoid ideation and psychoticism were higher in the stroke group, although the results were not statistically significant.^18^ Characteristics of all the included case series and quantitative studies are presented in online table 2 of the supplementary information.

### Treatment

No controlled studies that systematically investigated effectiveness of treatments for poststroke psychosis were found. However, information regarding treatment outcomes was available in 49 studies reporting a case study or case series and included 61 (23.1%) patients. A total of 48 of these studies reported some form of pharmacological treatment and intervention, specifically in the form of antipsychotic medication. Haloperidol and risperidone were the most common types of antipsychotic medications used to treat poststroke psychosis, which were prescribed to 13 (4.9%) and 12 (4.5%) of the patients, respectively. Other frequently prescribed types of antipsychotic medication included quetiapine and olanzapine, which were prescribed to 8 (3%) and 7 (2.7%) of patients, respectively. Psychological treatments were only reported in six of the studies and were provided to six patients (2.3%). Most often psychotherapy was provided in combination with pharmacological treatment. Two studies involved psychoeducation, either individually or with the patient’s family in addition to cognitive behaviour therapy, and one study involved sexual counselling. The remaining studies did not specify type of psychotherapy provided.

Treatment outcomes were reported in 58 studies reporting a case study or case series and included 82 patients (31%) and were categorised as complete resolution, partial resolution and no improvement (see online table 1 of the supplementary information). Complete resolution was defined as full resolution of the psychotic symptoms, partial resolution was defined as partial improvement or full resolution with periods of relapse and no improvement was defined as persistent psychotic symptoms. The most common outcome was complete resolution of psychotic symptoms, which was reported in 45 (17%) of patients. Partial resolution was reported in 27 patients (10.2%), and no improvement was reported in 10 patients (3.8%). Time to complete resolution was reported in 24 studies and included 40 patients (15.2%). The average time to complete resolution was 3.5 months.

### Long-term outcomes

Four of the included studies explored long-term outcomes of poststroke psychosis. One longitudinal study^15^ investigated the course of neuropsychiatric symptoms in geriatric patients admitted for stroke rehabilitation found that the presence of delusions and hallucinations changed less than 5% within the course of a year, suggesting that such symptoms remain fairly stable over time. The same study also found that patients who could not be discharged to independent living within 1 year experienced significantly more delusions and hallucinations compared with patients that could be discharged. van Almenkerk *et al*^19^ reported a cross-sectional study finding that a long-term consequence of stroke in relation to emotional and social well-being was experiencing pain, which was independently related to delusions.

A retrospective cohort study by Almeida and Xiao^8^ found that, compared with stroke patients without psychiatric disorders, patients with poststroke psychosis were 51% more likely to die during a 10-year follow-up period. Patients with poststroke psychosis also had the lowest survival rate compared with stroke survivors with other psychiatric disorders, with the most frequent cause of death being cardiovascular disease. Finally, a case series^20^ that compared patients with late-onset paranoia and paraphrenia found that patients with cerebral infarctions responded significantly worse to treatment. However, the same study reported that the same patients were given significantly less antipsychotic medication. Patients with cerebral infarctions were less isolated and more likely to have married compared with patients without infarctions.

### Other risk factors for poststroke psychosis

Previous psychiatric history was available in 51 studies reporting a case study or case series and included 115 (43.6%) patients. In a total of 106 (40.2%) patients, there was no psychiatric history. Among poststroke psychosis patients with a previous psychiatric history, alcohol misuse and depression were the most common, reported in seven (2.7%) patients and six (2.3%) patients, respectively. Additionally, one patient had a previous anxiety disorder, one patient had schizophrenia and one patient had a previous learning disability. Other risk factors reported included medical and lifestyle risk factors reported in 48 studies (including 134 patients; 50.8%). The most common medical risk factor was hypertension (72 patients; 27.3%), followed by hyperlipidaemia (36 patients; 13.6%) and diabetes mellitus (34 patients; 12.9%).

Two of the case series included in this review^9 10^ investigated risk factors for poststroke psychosis. One case series study reported hypertension, smoking, hyperlipidaemia, type 2 diabetes, hyperhomocysteinaemia and atherothrombosis as risk factors for the development of delusions after stroke.^9^ A further case series included comparisons with a small healthy control sample, reporting that patients with poststroke psychosis were more likely to have a previous psychiatric history compared with controls.^10^ One retrospective cohort study^21^ investigating psychotic symptoms in patients undergoing cardiac surgery found stroke to be an independent risk factor for developing psychotic symptoms.

### Quality assessment

Quality assessment of the case series and quantitative studies is presented in the online supplementary material. Studies were mostly of low quality, with the literature largely consisting of single cases, with only a few systematic studies.

## Discussion

We report the results of a preregistered systematic review on poststroke psychosis and report on presenting features, prevalence and incidence, treatment, lesion location risk factors and long-term outcomes.

### Clinical presentation

Previous literature has suggested poststroke psychosis is ‘rare’,^4^ although the meta-analytic summary presented here suggests that either delusions or hallucinations with poor insight occur in an estimated 4.86% of patients with postacute stroke. Prevalence is therefore likely greater than previously assumed, although we note the need for additional studies in this area, as the estimate reported here was based on a total of four studies, only three of which used a structured assessment of symptoms.

The average time between a stroke and the onset of psychosis or psychotic symptoms was reported in two studies, although with differing results. Almeida and Xiao^8^ reported an average time to onset for psychosis of 6.1 months over a follow-up period of 10 years. Focusing specifically on delusions after acute admission, Kumral and Oztürk^9^ reported an average time to onset of 2 days with a short duration but with presentations also including agitation, aggression, emotionalism, anxiety reactions and depression. Although only two patients are reported as having acute disorientation, given the other presenting features, acute admission population and use of clinical diagnosis, it is not clear to what extent they successfully screened out patients with fluctuating consciousness or confusional states when assessing for delusions, and we suspect Almeida and Xiao^8^ provide a better estimate of psychosis onset.

It is worth noting that studies on traumatic brain injury (TBI) have also reported a significant delay from point of insult to psychosis onset, with psychosis becoming more likely 2–3 years the index injury.^22^ This is additional evidence that although somatoparaphrenia and anosognosia are often classified as delusions, they appear aetiologically distinct from poststroke psychosis because they typically occur in the acute poststroke stage and quickly resolve.^23^ In contrast, poststroke psychosis (like post-TBI psychosis) seems often to occur after a significant delay, suggesting that each could be understood as arising from disruption to distinct stages of the recovery trajectory after brain insult.

A total of 18.1% of the patients in this review presented with haemorrhagic strokes, whereas 79.8% presented with ischaemic strokes. These findings are comparable with those found in the general stroke population, where haemorrhagic strokes normally account for approximately 20% of strokes and ischaemic strokes account for the largest proportion^24^ suggesting that stroke type is not a predictor of subsequent psychosis. The average age for the occurrence of poststroke psychosis was 66.6 years, and it occurred more often in men than in women. In terms of gender balance, these findings are in contrast to the wider stroke population, where women show a higher stroke prevalence.^25^ It is not clear why men might show a higher rate of poststroke psychosis, and this prevalence does not track the background risk factors for psychosis where men have a higher lifetime risk of developing primary psychotic disorders, such as schizophrenia,^26^ but women are over-represented in cases of late onset psychosis, which occur after the age of 60 years.^27^ Compared with poststroke depression, which has been more extensively researched, subsequent depression is more common in women. However, the interaction between gender and stroke risk is complex and involves historical, neurological and social risk factors.^28^ We expect a similar complex picture to occur in poststroke psychosis as more systematic evidence accumulates.

The majority of patients were found to have right hemisphere lesions, in line with a large body of evidence that suggests right hemisphere pathology is associated with a range of perceptual anomalies and pathologies of belief.^29^ The regions that were most affected included the right frontal, temporal and parietal regions, as well as the right caudate nucleus, supporting findings of previous case series on poststroke psychosis^10–12^ and mirroring findings in psychosis after TBI.^21^ However, it is worth bearing in mind that left hemisphere strokes are more likely to result in language and communication problems, which may impede the assessment of psychosis, and therefore these patients may be under-represented in the literature.^30^ It is also worth noting that these findings are largely based on clinical radiological assessments. To date, we are only aware of two studies that have used voxel-based lesion mapping across patients with stroke to generate maps of common lesions associated with psychosis. Devine *et al*^11^ examined lesion location in three patients with poststroke psychosis finding that damage to the right inferior frontal gyrus and connecting white matter tracts were common across patients. More recently, Darby *et al*^31^ used lesion network mapping in 17 stroke patients with misidentification delusions, identifying the left retrosplenial cortex as the only brain area functionally connected to all lesion sites, potentially highlighting its role in the aetiology of poststroke psychosis. Additional studies using these more systematic approaches are clearly needed to better clarify how damage to specific areas and neural circuits could lead to psychosis.

The most common presenting neurological signs and symptoms accompanying poststroke psychosis were left-sided weakness, headache and slurred speech. Others included left-sided neglect, left-sided decreased vision or blindness, left-sided facial weakness and confusion. Notably, these are all common presenting symptoms of stroke, and it is unclear to what extent they can be considered to be specific predictors of poststroke psychosis beyond indicating right hemisphere lesion.^32^ Importantly, however, a significant minority of patients were found to have otherwise ‘silent strokes’, where neurological signs were not obvious, not present or were subtle enough to be missed during referral to a psychiatrist for psychosis, suggesting that stroke may go undetected for a considerable amount of time. It is unclear to what extent the development of psychosis might affect the later chances of stroke detection, given that psychosis is typically associated with a significant delay in accessing treatment.^33^

The most common types of psychosis were, in order of frequency, delusional disorder, schizophrenia-like disorder and mood disorders with psychotic features. Notably, delusional disorder was more common than schizophrenia-like psychosis in this population, despite the fact that ‘non-organic’ delusional disorder has been found to have a relatively low lifetime prevalence in the general population.^34 35^ Evidence that delusional disorder and schizophrenia-like disorder are also the most common types of psychosis to follow after TBI^36^ suggests that more circumscribed lesions may produce less systematised, monothematic delusions that are less likely to be accompanied by hallucinations.

In terms of delusional theme, persecutory delusions were the most common, followed by delusional jealousy (Othello syndrome), reduplicative paramnesia and somatic delusions. Delusional jealousy seems to be relatively common here: although persecutory delusions and reduplicative paramnesia are also common after TBI, delusional jealousy is not as frequently reported.^36^ However, in comparison with its prevalence in schizophrenia and bipolar disorder, delusional jealousy seems to be more common in neurological disorders and neurodegenerative diseases for reasons that are still unclear.^37^

The majority of patients did not have a previous psychiatric history, although this was somewhat inconsistently reported across studies. Other risk factors included hypertension, hyperlipidaemia and diabetes, which are consistent with general risk factors for stroke.^38^

Diagnostic categories for poststroke psychosis are used inconsistently in the literature, and it is not clear which rating scales are most appropriate in this population, introducing a barrier to more systematic research in the area. Indeed, only 11 of the included studies applied diagnostic criteria, as specified by the Diagnostic and Statistical Manual of Mental Disorders and International Classification of Diseases, and only 12 studies included information about what psychosis assessment measures were used. This lack of structured assessment makes it difficult to draw more precise conclusions concerning differences in the presentation and severity of psychosis. Our review finds that clinicians have few options when faced with people in the clinic, and there is a need for a more structured assessment of psychosis.

### Treatment and outcome

No studies formally evaluated treatments for poststroke psychosis, but the available evidence suggests that antipsychotic medication is the most common form of treatment. Haloperidol and risperidone are reported as the most frequently prescribed, followed by quetiapine and olanzapine. It is worth noting that these types of treatments are linked to side-effect profiles that include dyskinesias and weight gain, and some, especially risperidone, are themselves a risk factor for developing stroke.^39^ It is therefore a cause for concern that so many individuals with poststroke psychosis are prescribed antipsychotic medication when no clinical trial has evaluated clinical effectiveness, safety or adverse effects. Our review identifies this as a research priority.

Psychological treatments were reported only in a few studies, indicating that psychological intervention as a treatment for poststroke psychosis remains largely unexplored. This is despite the fact that neuropsychological rehabilitation and psychological intervention are commonly used with people affected by stroke and psychosis, respectively, suggesting an opportunity for non-pharmacological management.

It is worth noting that the most common treatment outcome reported in the literature was complete resolution of poststroke psychosis, with the average time interval to complete resolution 3.5 months. These findings suggest that standard psychiatric management for poststroke psychosis is a reasonable approach. However, it remains unclear to what extent publication bias has played a role in presenting positive cases, how poststroke psychosis would resolve in the absence of treatment and what types of treatment are most effective.

Nevertheless, the evidence reviewed here suggests that long-term outcomes of poststroke psychosis are generally poor, indicating that patients with poststroke psychosis may have greater difficulties coping with the consequences of stroke and more likely to depend on assistance in their everyday lives compared with other stroke survivors. Furthermore, preliminary evidence suggests that patients with poststroke psychosis have an increased 10-year mortality risk.^8^ The reasons for this are currently unclear, and additional work is needed to understand to what extent this is due to the complicating effect of psychosis and to what extent due to the ongoing effects of background risk factors for psychosis itself. However, unhealthy lifestyle choices, antipsychotic side effects, suicide and physical illnesses, including cardiovascular illnesses, have all been found to contribute to excess mortality in primary psychosis,^40^ and we hypothesise that similar factors may affect those with poststroke psychosis.

### Limitations

Despite our systematic approach to this review, several limitations should be noted in terms of the findings and wider evidence base. The single most important limitation is the methodological quality of studies reviewed, which was generally poor. The majority were single cases, with group studies typically reporting small sample sizes. Moreover, only nine studies included a control group, and only one of the included studies was a prospective cohort study. In addition, the poor quality of reporting (in case studies particularly) meant that many key details needed to complete an adequate quality assessment were missing.

Given this, it is important to highlight that numerical summaries of published cases may be a poor guide to actual characteristics in the stroke population. In one instance, namely stroke type prevalence, the frequencies in published cases match estimates from epidemiological studies quite closely. However, it is likely that summaries of published case studies will be poorly representative of many aspects of poststroke psychosis, particularly, we suspect, regarding the type of delusion or hallucination, where rarer presentation may be more commonly reported in the literature, and treatment outcome, where patients with poorer outcome are likely to be under-represented. Currently, there are no systematic treatment studies, and this remains a marked deficiency in the literature and a significant barrier to evidence-based care. With this in mind, any characteristics of poststroke psychosis described in this review that are not echoed by systematic studies need to be treated, at best, as preliminary, until these studies are completed.

It is also worth noting that the inclusion criteria used in this current review was specifically designed to include true cases of psychosis (delusions and/or hallucinations with limited or absent insight). However, this meant that patients with poststroke hallucinations with full insight into the nature of their hallucinatory experience (hallucinosis) were not included, and it is not clear to what extent this group differs from patients with poststroke psychosis.

## Conclusions

We estimate that poststroke psychosis affects approximately 1 in 20 patients with stroke in the postacute stage, and one longer-term study^8^ suggests that this may increase to approximately 7% over subsequent years. Although high-quality studies are lacking to make a quantitative estimate, poststroke psychosis is regularly reported as being associated with particularly poor outcome. Considering the high prevalence of stroke in the population, poststroke psychosis should be common enough to study systematically. However, the lack of well-controlled group studies means there is only a limited evidence base to inform clinical decision making, and the completion of systematic studies needs to be made a research priority. The typical onset of poststroke psychosis may be several months after stroke suggesting a window for early detection and intervention. Research that identifies people at high risk of poststroke psychosis may lead to more effective treatment.

Although it is always possible to say that ‘more research is needed’ in the testing or development of interventions, the urgency is perhaps a little more apparent in the area of poststroke psychosis given that some of the most widely reported treatments (antipsychotics) can raise the risk of stroke. This also raises the question of whether the iatrogenic effects of these treatments contribute to the high mortality rates in poststroke psychosis. Understanding whether these adverse effects are present in a population who have already experienced stroke would seem an important step in terms of research most likely to have immediate benefit for clinical practice.

Currently, there are no structured assessments specific to poststroke psychosis and the validation of a psychosis assessment tool in poststroke psychosis would facilitate systematic studies in the area. However, a relatively straightforward improvement would be the standardised use and reporting of the relevant diagnostic categories.
