# Supplementary material for: Poststroke psychosis: a systematic review
Source: J Neurol Neurosurg Psychiatry. 2018 Jan 13;89(8):879–85. doi: 10.1136/jnnp-2017-317327 (PMC6204934; doi:10.1136/jnnp-2017-317327)
Supplement: Supplementary file 1 [file jnnp-2017-317327supp001.pdf]

Supplementary Material for

Post-stroke psychosis: a systematic review

Helle Stangeland, Vasiliki Orgeta, Vaughan Bell

Table of contents

Methods

Databases and search strategy 2

Full search strategy 2

Data extraction and quality assessment 3

Results

Reasons for exclusion 4

Meta-analysis of prevalence 5

Quality assessment of the included case series and quantitative studies 7

Characteristics of included single case reports 8

Characteristics of included case series and quantitative studies 17

Items selected for the assessment of the quality of the included group studies 24

References 27

Methods

Databases and search strategy

CINAHL, MEDLINE and PsychINFO were electronically searched from January 1975 to December 2016. We judged the 1st of January 1975 to be a natural starting date for the search, as studies published prior to this date are unlikely to involve neuroimaging techniques that provide critical information for this type of research, i.e. CT scans. The final search was completed on 9 December 2016. The search strategy was limited to only include studies published in English and studies assessing human participants.

Full search strategy

CINAHL (EBSCO), MEDLINE (OVID) and PsychINFO (OVID)

1. (((stroke or poststroke or post-stroke or cerebrovasc* or "cerebral vasc*" or CVA or apoplex* or SAH or ischemic* or ischaemic* or hemorrhage* or haemorrhage* or "transient ischemic attack" or "transient ischaemic attack" or TIA) and (psychosis or psychotic or delus* or hallucin* or paranoi* or schizophren*)) not dementia).mp.

2. limit 1 to (english language and humans and yr="1975 -Current")

MEDLINE (OVID) example

Search strategy

1. stroke/
2. poststroke/
3. post-stroke/
4. cerebrovasc$.tw.
5. cerebral vasc$.tw.
6. CVA/
7. apoplex$.tw.
8. SAH/
9. ischemic$.tw.
10. ischaemic$.tw.
11. hemorrhage$.tw.
12. haemorrhage$.tw.
13. ‘transient ischemic attack’/
14. ‘transient ischaemic attack’
15. TIA/
16. 1 or 2 or 3 or 4 or 5 or 6 or 7 or 8 or 9 or 10 or 11 or 12 or 13 or 14 or 15
17. psychosis/

18. psychotic/
19. delus$.tw.
20. hallucin$.tw.
21. paranoi$.tw.
22. schizophren$.tw.
23. 17 or 18 or 19 or 20 or 21 or 22
24. dementia/
25.16 and 23 not 24

Data extraction and quality assessment

Two reviewers independently screened the titles and abstracts of the literature identified by the search strategy described above, excluding studies that did not meet the inclusion criteria. During this process any study whose eligibility could not be determined after having reviewed the title and abstract was obtained via full text format, and included in the next stage of the search process. The full text of the remaining studies were then screened by the same reviewers and again excluded if they did not meet the inclusion criteria. Any uncertainty or disagreement about the studies at this stage was discussed between the two reviewers in order to reach consensus, or with the third author.

Finally, two reviewers used a standardised extraction form to extract relevant data from the included studies. Data was extracted with respect to study design, patient setting and patient characteristics, including age and number of participants, and whether or not the participants had been diagnosed with a psychiatric disorder prior to having a stroke. Moreover, information regarding each study's inclusion and exclusion criteria, comparison group or condition, and diagnostic assessment was extracted. Specifically, information about stroke subtype, lesion location, as well as time of onset and description of psychosis was obtained. If a study explored treatment interventions the method for assessing treatment outcomes was also extracted, as was each study's primary outcome and other outcomes. No authors were contacted for additional information.

Quality assessment was conducted using a revised version of the STROBE checklist for evaluating observational studies. Ten items were adapted from the STROBE checklist as there is currently no consensus about what assessment tool is appropriate to use for qualitative studies. Each item was scored either "Yes" or "No" by two reviewers and a global score was calculated for each item. The quality assessment is available below.

Results

Reasons for exclusion

After having screened the titles and abstracts of the studies identified by the search strategy, 198 were identified as potentially eligible. Out of these, 122 studies were excluded after having been obtained the full text, as they did not meet the inclusion criteria for the review. 41 of these studies were excluded because they did not meet the inclusion criteria for psychosis. Typically these studies involved non-psychotic hallucinatory symptoms due to sensory impairments, such as Charles Bonnet syndrome, or delusions relating to body ownership or body awareness, such as somatoparaphrenia and anasognosia. A total of 35 studies were excluded because the causation between stroke and psychosis was unclear, for example where patients were presenting with a comorbid neurodegenerative disease. A further 18 studies were excluded because they did not involve psychosis, and 13 studies were excluded due to unclear psychosis definitions, such as symptoms presented being representative of affective disorders rather than psychosis. We excluded 6 studies because they did not involve stroke pathology and 5 studies because they did not include CT or MRI scans to verify a diagnosis of stroke. Finally, 3 studies were excluded because they were reviews and did not report any original data, and 1 study was a personal account.

Meta-analysis of prevalence

Three studies reported prevalence of delusions and hallucinations in stroke patients. Kumral and Öztürk (2004) reported on delusions in a sample of acute stroke patients, Buijck et al (2011) reported prevalence rates for delusions and hallucination on admission and discharge in a sample of geriatric patients admitted for stroke rehabilitation, and van Almenkerk et al (2012) reported prevalence rates of delusions and hallucinations in a sample of institutionalized stroke patients. To estimate cross-sectional prevalence we only included prevalence at the point of admission from Buijck et al (2011).

It is worth noting differences in the included studies. Buijck et al (2011), van Almenkerk et al (2012) and Wong et al (2016) used the Neuropsychiatric Inventory Questionnaire to assess symptoms in patients admitted for stroke rehabilitation, or in the case of Wong et al (2016), patients admitted acutely but assessed 3-6 months after stroke and in the rehab phase, whereas Kumral and Öztürk (2004) reported ratings of delusions from clinical judgment based on DSM-IV criteria in patients admitted for acute stroke treatment.

Data from these studies were analysed using the *meta* package (version 4.8.4) for *R* statistics (version 3.4.2) on a 64-bit linux x86 platform. Freeman-Tukey double arcsine transformation was used to calculate the weighted summary proportion under the fixed and random effects models with normal approximation confidence internals for individual studies. The *R* analysis script and data have been made freely available on Open Science Framework archive for this analysis: https://osf.io/kzht6/

The formal test of heterogeneity for data included in the analysis was significant. Because of this, estimated prevalence was based on more conservative estimates from the random effects model throughout. Results from this meta-analysis are reported in Figure 2 of the main manuscript.

Because of the methodological differences in Kumral and Öztürk (2004) and concerns about adequate screening for patients with alterations in their levels of consciousness, not least because they various report patients with agitation and two specifically with confusional states, we also

report meta-analytic results with this study removed, using the same methods, in Figure S1 below, leading to very similar estimates of prevalence.

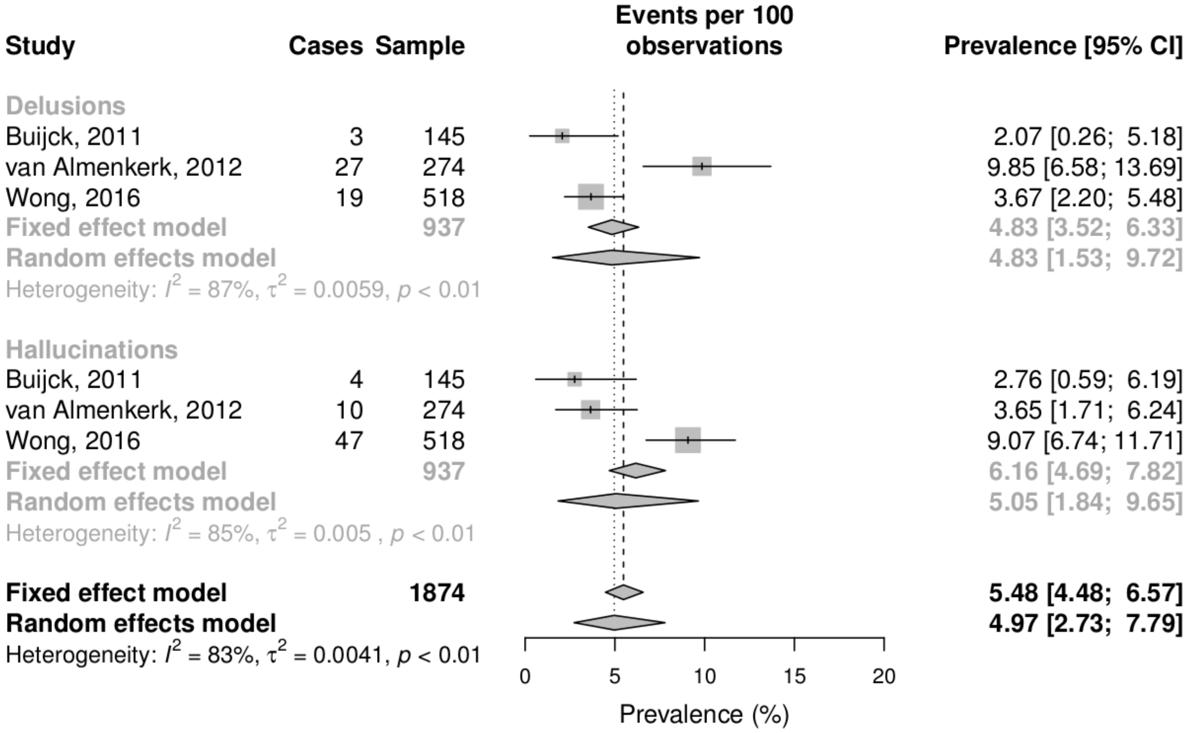

Figure S1. Forest plot of post-stroke delusion and hallucination prevalence with Kumral and Öztürk (2004) removed

Quality assessment of the case series and quantitative studies

All of the 18 studies included an adequate description of the setting and location of the studies, and specified the eligibility criteria and sources and methods for the selection of the participants. A total of 13 (72.2%) studies included definitions of psychosis as measured by diagnostic criteria, and 6 (33.3%) specified the diagnostic criteria used to diagnose stroke. In 11 (61.1%) of the studies stroke data were obtained from a patient or proxy, whereas in 7 (38.9%) stroke data was collected from a database. A total of 11 (61.1%) studies clearly defined their neuroimaging findings, and 3 (75%) of the eligible studies included a blinded investigator. We found that 17 (94.4%) of the studies adequately reported the number of participants at each stage of the study. All of the studies reported appropriate descriptive and outcome data. Finally, 6 (50%) of the eligible studies provided unadjusted estimates and their precision (e.g. 95% confidence interval) in their main results. Full results are displayed in Table 3.

Table 1. Characteristics of included single case reports

H = Haemorrhagic; I = Ischaemic; n/a = Not available; CBT* = Cognitive Behavioural Therapy

Reference	Age/Sex	Stroke	Psychosis type	Onset	Lesion	Treatment	Outcome
Akinci, Oncu & Topcular (2016)	61/M	I	Persecutory delusions and tactile hallucinations	n/a	Right temporo-parietal-occipital region	n/a	Partial resolution
Almeida et al. (2011)	20/F	H	Schizophrenia- like	1y	Right cerebellar hemisphere	Clozapine, valporate	Partial resolution
Anderson et al. (1998)	48/F	H	Erotomania	4y	Right fronto-parietal region	Risperidone, sertraline	n/a
Barboza et al. (2013)	54/F	I	Persecutory delusions	1y	Right fronto-temporo-parietal region	Quetiapine	Partial resolution
Bieganska & Janic (2015)	70/M	I	Paranoid delusions of reference and delusions of control	n/a	Right putamen, corona radiata and caudate nucleus	n/a	n/a
Bouckoms et al. (1986)	66/F	H	Capgras syndrome	n/a	Right frontal region	None	Partial resolution

Celik et al. (2004)	69/F	I	Mania with mood congruent psychotic symptoms	2d	Right temporo-parietal region	Carbamazepine	Partial resolution
Chae et al. (2006)	63/M	I	Othello syndrome	3.5y	Right fronto-parietal region and right basal ganglia	Quetiapine, zolpidem	Partial resolution
Chiu (1995)	78/F	H	Auditory and sensory hallucinations and secondary persecutory delusions	10d	Right occipital lobe and right superior-posterior parietal region	Haloperidol	Complete resolution
Crail-Melendez et al. (2013)	37/F	I	Schizophrenia-like	n/a	Right thalamus	Risperidone	Complete resolution
Das et al. (2005)	65/F	I	Erotomania	n/a	Fronto-temporal region, unspecified	Olanzapine and psycho-therapy	Partial resolution
Dayus & van den Broek (2000)	42/M	H	Stable delusional confabulations	n/a	Right caudate nucleus	Sulpiride, fluoxetine and self-monitoring training (SMT)	Partial resolution

Delgado & Bogousslavsky (2013)	48/F	I	Auditory distorteidolias	n/a	Bilateral thalamus (predominating right dorsomedial nucleus region)	None	Complete resolution
Dhasmana et al. (2010)	28/F	I	Psychosis, unspecified	1w	Left transverse sinus	None	Complete resolution
Floris et al. (2008)	70/M	H	Delusional parasitosis and visual hallucinations	5m	Left fronto-parietal region	Olanzapine	Partial resolution
Flynn et al. (1989)	67/M	I	Delusional parasitosis and auditory hallucinations	n/a	Right fronto-parietal region	Amoxapine	No improve-ment
Fonseca et al. (2013)	72/M	I	Cotard's syndrome	2m	Right fronto-temporo-parietal region	None	Complete resolution
Gnanavel (2013)	8/M	I	Paranoid delusions and auditory hallucinations	n/a	Left fronto-temporo-parietal region	Risperidone	Partial resolution
Hanihara et al. (2009)	64/F	I	Delusional parasitosis	3m	Right cerebellum and left thalamic region	Sulpiride	Partial resolution

Hinkebein et al. (2001)	67/M	H	Reduplicative paramnesia	2w	Right fronto-temporo-parietal region	Family psycho-education, CBT	Partial resolution
Hudson et al. (2000)	71/F	I	Fregoli syndrome and reduplicative paramnesia	1w	Right temporal region	n/a	n/a
Jocic et al. (1993)	67/M	I	Fregoli's syndrome and reduplicative paramnesia	15d	Right fronto-temporal region and right basal ganglia	Nortriptyline	Partial resolution
Kato et al. (2006)	64/F	I	Somatic delusions	4y	Right caudate nucleus	Antipsychotic drugs, unspecified	No improvement
Kitabayashi et al. (2006)	40/F	I	Schizophrenia-like	n/a	Right putamen, caudate and insula	Risperidone	Complete resolution
Klasen, Britton & Newman (1999)	12/M	I	Schizophrenia-like	n/a	Left basal ganglia region	Diazepam, chlorpromazine and trifluoperazine	Complete resolution
Lee et al. (2011)	69/M	n/a	Reduplicative paramnesia	2m	Right frontal region and right caudate nucleus	n/a	No improvement

Leeds et al. (2011)	71/F	I	Cotard's syndrome	n/a	Right thalamus	Antipsychotic drugs, unspecified	Complete resolution
Meguro et al. (2012)	77/M	I	Erotomania and Othello syndrome	n/a	Left head of the caudate nucleus	Risperidone, levomepromazine	Partial resolution
Mishra et al. (2008)	42/M	I	Paranoid delusions and visual hallucinations	2y	Bilateral occipital region	Phenytoin sodium, sodium valporate	n/a
Mittal & Khan (2010)	19/F	I	Paranoid delusions and auditory hallucinations	n/a	Left ventro-anterior thalamic nucleus	Haloperidol, olanzapine, risperidone and aspirin	1: Died from complications
Mobbs et al. (2001)	1: 34/M<br>2: 59/F	1: H<br>2: H	1: Thought disorder<br>2: Persecutory delusions	1: n/a<br>2: n/a	1: n/a<br>2: n/a	1: None<br>2: None	1: n/a<br>2: Complete resolution
Moriyama et al. (2007)	68/F	I	Fregoli syndrome	2m	Right temporo-occipital region	Risperidone	Complete resolution
Nagaratnam et al. (2000)	1: 69/F<br>2: 65/F	1: I<br>2: I	1: Delusional parasitosis<br>2: Delusional parasitosis	1: 6m<br>2: 3w	1: Left temporo-occipital region<br>2: Left temporo-occipital region	n/a	n/a

Narumoto et al. (2005)	55/F	I	Persecutory delusions and visual hallucinations	n/a	Bilateral head of the caudate nucleus	Haloperidol	Partial resolution
Narumoto et al. (2006)	82/F	I	Delusional parasitosis and visual hallucinations	1m	Right temporo-parietal region	Risperidone	Complete resolution
Neetesh et al. (2012)	49/F	H	Othello syndrome	2m	Right basal ganglia	Risperidone	Complete resolution
Ohara et al. (2006)	56/F	n/a	Auditory hallucinations	4m	Left temporal and parietal region	Haloperidol	Complete resolution
Ortiz et al. (2004)	30/M	I	Depression with mood congruent psychotic symptoms	n/a	Left para-hippocampal gyrus	Clomipramine, citalopram, venlafaxine, haloperidol, risperidone, clozapine, olanzapine	Partial resolution
Pakalnis et al. (1987)	52/F	I	Capgras syndrome and visual hallucinations	1d	Right parieto-occipital region	Haloperidol	Complete resolution

Peckins, Khorashadi & Wolpow (2016)	88/F	I	Reduplicative paramnesia	8d	Medial right thalamus	n/a	Complete resolution
Perez et al. (2014)	73/F	H	Cotard's syndrome	n/a	Right fronto-parietal region	Levetiracetam	Complete resolution
Richardson et al. (1991)	68/M	I	Othello syndrome	2m	Right fronto-temporo-parietal region	Haloperidol, phenytoin, and sexual counseling	No improve-ment
Richardson (1992)	64/M	I	Unspecified delusions and visual hallucinations	n/a	Right temporo-parieto-occipital region and left cerebellum	Haloperidol	Partial resolution
Rocha et al. (2014)	1: 65/M<br>2: 69/M	1: I<br>2: I	1: Othello syndrome and persecutory delusions<br>2: Othello syndrome	1: 1w<br>2: 1d	1: Right temporo-parieto-insular region<br>2: Right temporo-parieto-insular region	1: Fluvoxamine, chlorpromazine<br>2: Quetiapine, sertraline	1: Complete resolution<br>2: Complete resolution

Routh & Hill (2014)	83/M	H	Mania with paranoid delusions	n/a	Right posterior thalamus region	Olanzapine	Complete resolution
Santos et al. (2009)	1: 67/M<br>2: 49/M	1: I<br>2: I	1: Delusions of self-reference and persecutory delusions<br>2: Gustatory hallucinations and persecutory delusions	1: n/a<br>2: n/a	1: Right caudate nucleus<br>2: Left thalamus	1: Quetiapine, benzodiazepines, unspecified<br>2: Quetiapine and psychotherapy	1: Complete resolution<br>2: Complete resolution
Schnider et al. (1993)	54/M	n/a	Mania with mood congruent psychotic symptoms	n/a	Right temporo-parieto-occipital region	n/a	Partial resolution
Small (2016)	57/F	I	Visual and tactile hallucinations	2y	Pontine region	Quetiapine	Complete resolution
Sottile et al. (2015)	30/F	I	Capgras syndrome and Cotard's syndrome	n/a	Left temporo-parietal region	Olanzapine and psychotherapy	Complete resolution
Spiegel et al. (2008)	54/F	I	Capgras syndrome	1w	Right frontal region	Mirtazepine, ziprasidone	Partial resolution
Suhr et al. (1998)	52/F	I	Unspecified delusions and auditory hallucinations	10y	Right temporo-parieto-occipital region	Phenytoin, psychoeducation and CBT*	Partial resolution

Thiel et al. (2014)	70/F	H	Capgras syndrome	n/a	Right frontal region	n/a	n/a
Villarejo et al. (2011)	90/M	I	Mirrored-self misidentification and reduplicative paramnesia	1w	Right frontal region	None	No improvement
Westlake & Weeks (1999)	20/F	H	Depression with Othello syndrome	5y	Right parietal and frontal region	Paroxetine	Partial resolution
Wong et al. (1997)	72/M	I	Othello syndrome	1d	Right caudate nucleus and putamen	Nortriptyline	Partial resolution
Wunderlich et al. (2000)	24/F	I	Visual hallucinations	2w	Parietal-occipital cortex	None	Complete resolution
Yildiz et al. (2007)	56/M	H	Schizophrenia-like	6m	Left fronto-parietal region	Diazepam and haloperidol	Partial resolution
Zimnitzky et al. (1996)	19/M	I	Schizophrenia-like	n/a	n/a	Risperidone	Partial resolution

Table 2. Characteristics of included case series and quantitative studies

NPS* = Neuropsychiatric symptoms; n/a = Not available; RC = retrospective cohort study; PC = prospective cohort study; CS = case series; CrS = cross sectional study

Reference	Study design	Setting	Sample	Exclusion	Comparator	Primary outcome
Almeida (2007)	RC	Community, Australia	Stroke survivors with a first ever stroke in 1990 (N = 1008)	Previous psychiatric disorder	Matched controls without NPS* (N = 287)	Incidence and 10-year mortality of NPS* after stroke
Buijck et al. (2012)	PC	Nursing homes, The Netherlands	Geriatric patients admitted for stroke rehabilitation (N = 145)	Unwillingness to give informed consent, legally incapable, expected to be discharged within 2 weeks, critically ill	n/a	Prevalence and course of NPS* after stroke

Devine et al. (2014)	CS	Hospital, London	Patients with post-stroke psychosis (N = 3)	n/a	Patients with similar anatomical damage (N = 9)	To identify the neuroanatomical basis of psychosis following stroke
Flint et al. (1991)	CS	Hospital, Canada	Patients with late-onset paraphrenia (N = 21)	Previous psychiatric history of psychosis or affective disorders, comorbid neurological disease	Patients with late-onset paranoia (N = 12)	To determine the course of late-onset paranoia compared to paraphrenia
Giltay et al. (2006)	RC	Hospital, The Netherlands	Patients undergoing cardiac surgery (N = 8139)	n/a	Patients without psychotic symptoms (N = 7971)	Presence of psychotic symptoms after cardiac surgery

Hoffmann (2008)	CS	Stroke registry, United States	Isolated right temporal stroke patients (N = 5)	Extratemporal involvement by the lesion, coma, cerebral trauma, encephalopathy, substance abuse, neurodegenerative disease, medication related effects	Isolated left temporal stroke patients (N= 5)	Prevalence of temporal and frontal cognitive and neuropsychiatric syndromes in isolated right and left stroke patients
Kumral et al. (2004)	CS	Hospital, Turkey	First ever stroke patients with delusions (N = 15)	Previous psychiatric disorder, substance abuse, transient and intermittent behavioural abnormality after a seizure	n/a	To define delusional themes and anatomical patterns of delusions after stroke

Levine et al. (1984)	CS	Hospital, United States	Stroke patients with right cerebral infarcts and delusions (N = 9)	Comorbid neurological disease, medication related effects, congestive heart failure, respiratory failure	Stroke patients with right cerebral infarcts without delusions (N = 16)	To identify the nature and causes of delusions after infarction of the right cerebral hemisphere
McMurtray et al. (2008)	CS	Veterans memory disorder clinic, United States	Patients with content-specific delusions after right caudate stroke (N = 8)	Previous psychiatric and medical conditions that could affect cognition, medication related effects	Matched controls without stroke or delusions (N = 8)	Association with prefrontal hypometabolism

Murai et al. (1997)	CS	Hospital, Japan	Patients with focal brain damage and reduplicative paramnesia (N = 6)	No CT or MRI, previous psychiatric disorder, severe communication disturbances	n/a	To identify the prevalence and course of reduplicative paramnesia
Price & Mesulam (1985)	CS	Hospital, United States	Right-handed patients with right hemisphere infarcts and psychosis (N = 5)	n/a	n/a	Clinical features of psychosis due to cerebrovascular disease
Rabins et al. (1991)	CS	Hospital, United States	Patients with post-stroke psychosis (N = 5)	Previous psychiatric history of psychosis	Patients with the same lesion size and location without post-stroke psychosis (N = 5)	Risk factors for developing post-stroke psychosis

Ramirez-Bermudez, Espinola-Nadurille & Loza-Taylor (2010)	CS	Hospital, Mexico	Patients with neurological disease and delusional parasitosis (N = 4)	Patients without neurological disease	n/a	Clinical features and response to treatment
Su et al. (2001)	CS	Hospital, Taiwan	Patients with delusional disorder due to cerebrovascular disease (N = 7)	Previous psychiatric history or other non-vascular cortical/ subcortical neurological disorders	n/a	Clinical features and MRI characteristics of delusional disorder due to cerebrovascular disease
van Almenkerk et al. (2012)	CrS	Nursing homes, The Netherlands	Stroke patients admitted for stroke rehabilitation (N = 274)	Incomplete questionnaires	n/a	Status of functioning and stroke characteristics

van Almenkerk et al. (2015)	CrS	Nursing homes, The Netherlands	Stroke patients admitted for stroke rehabilitation (N = 274)	Incomplete questionnaires	n/a	Prevalence of pain and its relation to emotional distress and low social engagement
Wong et al. (2016)	CrS	Hospital, Hong Kong	TIA and stroke patients 3–6 months post index admission (N = 518)	History of dementia, not able to complete cognitive testing	n/a	Neuropsychiatric Inventory, cognitive function, amyloid imaging
Zhang et al. (2015)	RC	Hospital, China	Ischaemic stroke patients one year after hemiplegia (N = 90)	n/a	Matched controls without stroke (N = 90)	To measure psychological distress, social support and medication adherence

Table 3. Items selected for the assessment of the quality of the included group studies

**Methods**	
Setting	Description of the setting, locations, and relevant dates, including periods of recruitment, exposure, follow-up, and data collection.
Participants	The eligibility criteria, and the sources and methods of selection of participants. For matched studies, matching criteria and the number of controls per case.
Variables	Definition of psychosis and stroke (including diagnostic criteria, if applicable).
Data source and measurement	a. Method for collecting stroke data (directly from the patient or database).<br>b. Clearly defined CT/MRI measures.<br>c. Investigator blinded to patient status.
**Results**	
Participants	Report numbers of individuals at each stage of study—eg numbers potentially eligible, examined for eligibility, confirmed eligible, included in the study, completing follow-up, and analysed
Descriptive data	Give characteristics of study participants (eg demographic, clinical, social) and information on exposures and potential confounders.
Outcome data	Report numbers of outcome events or summary measures.
Main results	Give unadjusted estimates and, if applicable, confounder-adjusted estimates and their precision (eg, 95% confidence interval). Make clear which confounders were adjusted for and why they were included.

			**Data sources and measurement**							
**Studies**	**Setting and location**	**Sources and methods for selection of participants**	**Psychosis and stroke measures**	**Method for collecting stroke data**	**CT and MRI measures**	**Investigator blinded**	**Participants**	**Descriptive data**	**Outcome data**	**Main results**
Almeida (2007)	Yes	Yes	P: Yes<br>S: Yes	Database	No	N/A	Yes	Yes	Yes	Yes
Buijck (2012)	Yes	Yes	P: Yes<br>S: No	Patient	No	N/A	Yes	Yes	Yes	N/A
Devine (2014)	Yes	Yes	P :No<br>S: No	Database	Yes	N/A	Yes	Yes	Yes	N/A
Flint (1991)	Yes	Yes	P: Yes<br>S: No	Patient	Yes	Yes	Yes	Yes	Yes	No
Giltay et al. (2006)	Yes	Yes	P: No<br>S: No	Database	No	N/A	Yes	Yes	Yes	Yes
Hoffmann (2008)	Yes	Yes	P: Yes<br>S: Yes	Database	Yes	Yes	Yes	Yes	Yes	Yes
Kumral (2004)	Yes	Yes	P: Yes<br>S: Yes	Patient	Yes	N/A	Yes	Yes	Yes	No
Levine (1984)	Yes	Yes	P: No<br>S: No	Patient	No	N/A	Yes	Yes	Yes	No
McMurtray (2008)	Yes	Yes	P: No<br>S: Yes	Database	No	N/A	Yes	Yes	Yes	No
Murai (1997)	Yes	Yes	P: Yes<br>S: No	Patient	Yes	No	Yes	Yes	Yes	N/A
Price & Mesulam (1985)	Yes	Yes	P: No<br>S: No	Patient	Yes	N/A	Yes	Yes	Yes	N/A
Rabins (1991)	Yes	Yes	P: Yes<br>S: No	Patient	Yes	Yes	Yes	Yes	Yes	No

Ramirez-Bermudez, Espinola-Nadurille & Loza-Taylor (2010)	Yes	Yes	P: Yes<br>S: No	Database	Yes	N/A	Yes	Yes	Yes	N/A
Su et al. (2001)	Yes	Yes	P: Yes<br>S: No	Database	Yes	N/A	Yes	Yes	Yes	N/A
van Almenkerk (2012)	Yes	Yes	P: Yes<br>S: No	Proxy	No	N/A	Yes	Yes	Yes	Yes
van Almenkerk (2015)	Yes	Yes	P: Yes<br>S: No	Proxy	No	N/A	Yes	Yes	Yes	Yes
Wong et al. (2016)	Yes	Yes	P: Yes<br>S: Yes	Patient	Yes	N/A	Yes	Yes	Yes	Yes
Zhang et al. (2015)	Yes	Yes	P: Yes<br>S: Yes	Patient	Yes	N/A	No	Yes	Yes	No
**Total (%)**	100	100	P: 72.2<br>S: 33.3	Database: 38.9<br>Patient/ proxy: 61.1	61.1	75	94.4	100	100	50

References

Akinci E, Oncu F, Topcular B. Tactile hallucination and delusion following acute stroke: a case report. Dusunen Adam. 2016;29(1),79.

Almeida J, Serrao EM, Almeida AT, Afonso JG. Effective treatment with clozapine and valproate for refractory schizophrenia-like psychosis after cerebellar hemorrhage. Clinical neuropharmacology. 2011;34(3):131-132.

Almeida OP, Xiao J. Mortality associated with incident mental health disorders after stroke. Australian and New Zealand Journal of Psychiatry. 2007;41(3):274-281.

Anderson CA, Camp J, Filley CM. Erotomania after aneurysmal subarachnoid hemorrhage. The Journal of neuropsychiatry and clinical neurosciences. 1998.

Barboza RB, De Freitas GR, Tovar-Moll F, Fontenelle LF. Delayed-onset post-stroke delusional disorder: A case report. Behavioural neurology. 2013;27(3):287-291.

Biegańska JM, Janik B. Usefulness of diagnostic models in the process of qualifying thought contents as delusional: case analysis. Archives of Psychiatry and Psychotherapy. 2015;2,5-12.

Bouckoms A, Martuza R, Henderson M. Capgras syndrome with subarachnoid hemorrhage. The Journal of nervous and mental disease. 1986;174(8):484-488.

Buijck BI, Zuidema SU, Eijk M, Geurts AC, Koopmans RT. Neuropsychiatric symptoms in geriatric patients admitted to skilled nursing facilities in nursing homes for rehabilitation after stroke: a longitudinal multicenter study. International journal of geriatric psychiatry. 2011;27(7): 734-741.

Celik Y, Erdogan E, Tuglu C, Utku U. Post-stroke mania in late life due to right temporoparietal infarction. Psychiatry and clinical neurosciences. 2004;58(4),446-447.

Chae BJ, Kang BJ. Quetiapine for hypersexuality and delusional jealousy after stroke. Journal of clinical psychopharmacology. 2006;26(3):331-332.

Chiu HF. Poststroke psychosis. The British journal of clinical practice. 1995;49(5), 277- 277.

Crail-Melendez D, Atriano-Mendieta C, Carrillo-Meza R, Ramirez-Bermudez J. Schizophrenia-like psychosis associated with right lacunar thalamic infarct. Neurocase. 2013;19(1):22-26.

Das A, Mathur S, Orrell M. Erotomania secondary to cerebrovascular accident in frontotemporal impairment. Irish journal of psychological medicine. 2005;22(03):113-114.

Dayus B, Van den Broek MD. Treatment of stable delusional confabulations using self- monitoring training. Neuropsychological Rehabilitation. 2000;10(4), 415-427.

Delgado MG, Bogousslavsky J. ‘Distorteidolias'-Fantastic Perceptive Distortion. European neurology. 2013;70(1-2), 6-9.

Devine MJ, Bentley P, Jones B, Hotton G, Greenwood RJ, Jenkins, et al. The role of the right inferior frontal gyrus in the pathogenesis of post-stroke psychosis. Journal of neurology. 2014;261(3):600-603.

Dhasmana DJ, Brockington IF, Roberts A. Post-partum transverse sinus presenting as acute psychosis. Archives of women's mental health. 2010;13(4):365-367.
Flint AJ, Rifat SL, Eastwood MR. Late-onset paranoia: Distinct from paraphrenia?. International Journal of Geriatric Psychiatry. 1991;6(2):103-109.

Floris G, Cannas A, Melis M, Solla P, Marrosu MG. Pathological gambling, delusional parasitosis and adipsia as a post-haemorrhagic syndrome: a case report. Neurocase. 2008;14(5):385-389.

Flynn FG, Cummings JL, Scheibel J, Wirshing W. Monosymptomatic delusions of parasitosis associated with ischemic cerebrovascular disease. Journal of geriatric psychiatry and neurology. 1989;2(3):134-139.

Fonseca AC, Pinho e Melo T, Ferro JM. Cotard delusion after stroke. European Journal of Neurology. 2013;20(7):e98-e99.

Giltay EJ, Huijskes RV, Kho KH, Blansjaar BA, Rosseel PM. Psychotic symptoms in patients undergoing coronary artery bypass grafting and heart valve operation. European journal of cardio-thoracic surgery. 2006;30(1),140-147.

Gnanavel S. Poststroke psychosis in an 8-year-old child with moyamoya disease. BMJ case reports. 2013;bcr2013201771.

Hanihara T, Takahashi T, Washizuka S, Ogihara T, Kobayashi M. Delusion of oral parasitosis and thalamic pain syndrome. Psychosomatics. 2009;50(5):534-537.

Hinkebein JH, Callahan CD, Gelber D. Reduplicative paramnesia: Rehabilitation of content-specific delusion after brain injury. Rehabilitation Psychology. 2001;46(1):75.

Hoffmann M. Isolated right temporal lobe stroke patients present with Geschwind Gastaut syndrome, frontal network syndrome and delusional misidentification syndromes. Behavioural neurology. 2008;20(3-4):83-89.

Hudson AJ, Grace GM. Misidentification syndromes related to face specific area in the fusiform gyrus. Journal of Neurology, Neurosurgery & Psychiatry. 2000;69(5):645-648.

Jocic Z, Staton RD. Reduplication after right middle cerebral artery infarction. Brain and cognition. 1993;23(2):222-230.

Kato Y, Muramatsu T, Kato M, Shibukawa Y, Shintani M, Yoshino F. Cortical reorganization and somatic delusional psychosis: a magnetoencephalographic study. Psychiatry Research: Neuroimaging. 2006;146(1):91-95.

Kitabayashi Y, Narumoto J, Otakara C, Hyungin C, Fukui K, Yamada K. Schizophrenia-like psychosis following right putaminal infarction. The Journal of neuropsychiatry and clinical neurosciences. 2006;18(4):561.

Klasen H, Britton J, Newman M. Moyamoya disease in a 12-year-old Caucasian boy presenting with acute transient psychosis. European child & adolescent psychiatry. 1999;8(2), 149-153.

Kumral E, Öztürk Ö. Delusional state following acute stroke. Neurology. 2004;62(1):110-113.

Lee K, Shinbo M, Kanai H, Nagumo Y. Reduplicative paramnesia after a right frontal lesion. Cognitive and Behavioral Neurology. 2011;24(1):35-39.

Leeds L, Srinivasan J. Case Report: an unusual case of post-stroke psychosis in a 71-year-old lady with a learning disability. British Journal of Learning Disabilities. 2011;39(4):292-294.

Levine DN, Grek A. The anatomic basis of delusions after right cerebral infarction. Neurology. 1984;34(5):577-577.

McMurtray AM, Sultzer DL, Monserratt L, Yeo T, Mendez MF. Content-specific delusions from right caudate lacunar stroke: association with prefrontal hypometabolism. The journal of psychiatry and clinical neurosciences. 2008;20(1):62-67.

Meguro K, Meguro M, Akanuma K. Recurrent delusional ideas due to left caudate head infarction, without dementia. Psychogeriatrics. 2012;12(1):58-61.

Mishra NK, Hastak S. Poststroke hallucination delusion syndrome. The Journal of neuropsychiatry and clinical neurosciences. 2007;20(1):116-116.

Mittal M, Khan S. Starvation causes acute psychosis due to anterior thalamic infarction. Southern medical journal. 2010;103(7), 701-703.

Mobbs RJ, Chandran KN, Newcombe RL. Psychiatric presentation of aneurysmal subarachnoid haemorrhage. ANZ journal of surgery. 2001;71(1):69-70.

Moriyama Y, Muramatsu T, Kato M, Mimura M, Akiyama T, Kashima H. Fregoli Syndrome Accompanied with Prosopagnosia in a Woman with a 40-year History of Schizophrenia. Keio journal of medicine. 2007;56(4): 130.

Murai T, Toichi M, Sengoku A, Miyoshi K, Morimune S. Reduplicative paramnesia in patients with focal brain damage. Cognitive and Behavioral Neurology. 1997;10(3):190-196.

Nagaratnam N, O'Neile L. Delusional parasitosis following occipito-temporal cerebral infarction. General hospital psychiatry. 2000;22(2):129-132.

Narumoto J, Matsushima N, Oka S, Shimizu H, Kooguchi Y, Kitabayashi Y, et al. Neurobehavioral changes associated with bilateral caudate nucleus infarctions. Psychiatry and clinical neurosciences. 2005;59(1):109-110.

Narumoto J, Ueda H, Tsuchida H, Yamashita T, Kitabayashi Y, Fukui K. Regional cerebral blood flow changes in a patient with delusional parasitosis before and after successful treatment with risperidone: a case report. Progress in Neuro-Psychopharmacology and Biological Psychiatry. 2006;30(4):737-740.

Neetesh B, Yogaratnam J. Organic Othello Syndrome Following a Stroke: A Rare Complication. German Journal of Psychiatry. 2012;15(1):41-43.

Ohara, T, Monji A, Onitsuka T, Maekawa T, Hirano Y, Hirano S, et al. Interictal psychosis after stroke with forced normalization. The Journal of neuropsychiatry and clinical neurosciences. 2006;18(4),557-559.

Ortiz N, Annoni JM, Trojan D, Alberque C, Eytan A. Persistent severe depressive episode with mood-congruent psychotic features associated with left temporal ischemia. Cognitive and behavioral neurology. 2004;17(3),157-162.

Pakalnis A, Drake ME, Kellum JB. Right parieto-occipital lacunar infarction with agitation, hallucinations, and delusions. Psychosomatics. 1987;28(2):95-96.

Peckins CS, Khorashadi L, Wolpow ER. A Case of Reduplicative Paramnesia for Home. Cognitive and Behavioral Neurology. 2016;29(3),150-157.

Perez DL, Fuchs BH, Epstein J. A case of cotard syndrome in a woman with a right subdural hemorrhage. The Journal of neuropsychiatry and clinical neurosciences. 2013;26(1):E29-30.

Price BH, Mesulam M. Psychiatric manifestations of right hemisphere infarctions. The Journal of nervous and mental disease. 1985;173(10), 610-614.

Rabins PV, Starkstein SE, Robinson RG. Risk factors for developing atypical (schizophreniform) psychosis following stroke. The Journal of neuropsychiatry and clinical neurosciences. 1991;3(1):6-9.

Ramirez-Bermudez J, Espinola-Nadurille M, Loza-Taylor N. Delusional parasitosis in neurological patients. General hospital psychiatry. 2010;32(3),294-299.

Richardson ED, Malloy PF, Grace J. Othello syndrome secondary to right cerebrovascular infarction. Journal of geriatric psychiatry and neurology. 1991;4(3):160-165.

Richardson JK. Psychotic Behavior After Right Hemispheric Accident: A Case Report. Archives of physical medicine and rehabilitation. 1992;73:381-384.

Rocha S, Pinho J, Ferreira C, Machado Á. Othello Syndrome After Cerebrovascular Infarction. The Journal of neuropsychiatry and clinical neurosciences. 2014;26(3):E1-E2.

Routh R, Hill A. Post-stroke mania: a rare but treatable presentation. Progress in Neurology and Psychiatry. 2014;18(1), 24-25.

Santos S, Alberti O, Corbalán T, Cortina MT. Stroke-psychosis. Description of two cases. Actas Esp Psiquiatr. 2009;37(4):240-242.

Schnider A, Regard M, Benson DF, Landis T. Effects of a right-hemisphere stroke on an artist's performance. Cognitive and Behavioral Neurology. 1993;6(4), 249-255.

Small G. Progressive motor deficits and psychosis after stroke: a case presentation. Journal of Neuroscience Nursing. 2016;48(2), 68-70.

Sottile F, Bonanno L, Finzi G, Ascenti G, Marino S, Bramanti P, et al. Cotard and Capgras Syndrome after Ischemic Stroke. Journal of Stroke and Cerebrovascular Diseases. 2015;24(4):e103-e104.

Spiegel DR, Laroia R, Samuels D. A possible case of Capgras syndrome after a right anterior cerebral artery cerebrovascular accident treated successfully with mirtazapine. The Journal of neuropsychiatry and clinical neurosciences. 2007;20(4):494-494.

Su KP, Hsu CY, Hsieh SC, Shen WW. Magnetic resonance imaging findings in patients with delusional disorder due to diffuse cerebrovascular disease: a report of seven cases. Psychiatry and clinical neurosciences. 2001;55(2), 121-126.

Suhr JA, Anderson SW. Behavioral management of chronic hallucinations and delusions following right middle cerebral artery stroke. Psychotherapy: Theory, Research, Practice, Training. 1998;35(4):464.

Thiel CM, Studte S, Hildebrandt H, Huster R, Weerda R. When a loved one feels unfamiliar: a case study on the neural basis of Capgras delusion. Cortex. 2014;52,75-85.

van Almenkerk S, Depla MF, Smalbrugge M, Eefsting JA, Hertogh CM. Institutionalized stroke patients: status of functioning of an under researched population. Journal of the American Medical Directors Association. 2012;13(7):634-639.

van Almenkerk S, Depla MF, Smalbrugge M, Eefsting JA, Hertogh CM. Pain among institutionalized stroke patients and its relation to emotional distress and social engagement. International journal of geriatric psychiatry. 2015;30(10),1023-1031.

Villarejo A, Martin VP, Moreno-Ramos T, Camacho-Salas A, Porta-Etessam J, Bermejo- Pareja, F. Mirrored-self misidentification in a patient without dementia: evidence for right hemispheric and bifrontal damage. Neurocase. 2011;17(3):276-284.

Westlake RJ, Weeks SM. Pathological jealousy appearing after cerebrovascular infarction in a 25-year-old woman. Australian and New Zealand journal of psychiatry. 1999;33(1),105-107.

Wong A, Lau AY, Yang J, Wang Z, Liu W, Lam BY, Au L, Shi L, Wang D, Chu WC, Xiong YY, Lo ES, Law LS, Leung TW, Lam LC, Chan AY, Soo YO, Leung EY, Wong LK, Mok VC. Neuropsychiatric Symptom Clusters in Stroke and Transient Ischemic Attack by Cognitive Status and Stroke Subtype: Frequency and Relationships with Vascular Lesions, Brain Atrophy and Amyloid. PLoS One. 2016;11(9):e0162846.

Wong AH, Rosemary Meier HM. Case report: Delusional jealousy following right-sided cerebral infarct. Neurocase. 1997;3(5):391-394.

Wunderlich G, Suchan B, Volkmann J, Herzog H, Hömberg V, Seitz RJ. Visual hallucinations in recovery from cortical blindness: Imaging correlates. Archives of Neurology. 2000;57(4), 561-565.

Yildiz M, Pflueger M, Riecher-Rössler A, Berger G. Aneurysms of pericallosal cerebral artery haemorrhage with consecutive psychosis. The Australian and New Zealand journal of psychiatry. 2007;41(6):554-554.

Zhang H, Qian HZ, Meng SQ, Shu M, Gao YZ, Xu Y, et al. Psychological distress, social support and medication adherence in patients with ischemic stroke in the mainland of China. Journal of Huazhong University of Science and Technology [Medical Sciences]. 2015;35(3),405-410.

Zimnitsky BM, DeMaso DR, Steingard RJ. Use of risperidone in psychotic disorder following ischemic brain damage. Journal of child and adolescent psychopharmacology. 1996;6(1):75-7.
